# Early-life skin microbiota in hospitalized preterm and full-term infants

**DOI:** 10.1186/s40168-018-0486-4

**Published:** 2018-05-31

**Authors:** Noelle E. Younge, Félix Araújo-Pérez, Debra Brandon, Patrick C. Seed

**Affiliations:** 1 Department of Pediatrics, Duke University, Durham, NC USA; 2 Department of Pediatrics, Northwestern University, 310 E. Superior, Morton 4-685, Chicago, IL 60611 USA; 3 Duke University School of Nursing, Durham, NC USA

**Keywords:** Cutaneous, Microbiome, Neonate, Neonatal intensive care unit, *Staphylococcus*, *Escherichia*

## Abstract

**Background:**

The infant skin microbiota may serve as a reservoir of bacteria that contribute to neonatal infections and stimulate local and systemic immune development. The objectives of our study were to characterize the skin microbiota of preterm and full-term infants during their birth hospitalization and describe its relationship to the microbiota of other body sites and the hospital environment.

**Results:**

We conducted a cross-sectional study of 129 infants, including 40 preterm and 89 full-term infants. Samples were collected from five sites: the forehead and posterior auricular scalp (skin upper body); the periumbilical region, inguinal folds, and upper thighs (skin lower body); the oral cavity; the infant’s immediate environment; and stool. *Staphylococcus*, *Streptococcus*, *Enterococcus*, and enteric Gram-negative bacteria including *Escherichia* and *Enterobacter* dominated the skin microbiota. The preterm infant microbiota at multiple sites had lower alpha diversity and greater enrichment with *Staphylococcus* and *Escherichia* than the microbiota of comparable sites in full-term infants. The community structure was highly variable among individuals but differed significantly by body site, postnatal age, and gestational age. Source tracking indicated that each body site both contributed to and received microbiota from other body sites and the hospital environment.

**Conclusion:**

The skin microbiota of preterm and full-term infants varied across individuals, by body site, and by the infant’s developmental stage. The skin harbored many organisms that are common pathogens in hospitalized infants. Bacterial source tracking suggests that microbiota are commonly exchanged across body sites and the hospital environment as microbial communities mature in infancy.

**Electronic supplementary material:**

The online version of this article (10.1186/s40168-018-0486-4) contains supplementary material, which is available to authorized users.

## Background

After birth, the infant’s skin and mucosal surfaces are exposed to a variety of maternal and environmental microbes that may colonize the newborn. While our understanding of the development of the fecal microbiome in infancy has expanded greatly over the past decade, acquisition and succession of the skin microbiota is less well-studied. The skin undergoes dynamic structural and functional changes in infancy that may influence the development of the skin microbiome, including shifts in pH, water content, transepidermal water loss, and sebum production [1]. The extent to which skin maturation, clinical factors, and environmental exposures shape the neonatal skin microbiome is not well understood. In preterm infants, many invasive infections are caused by bacteria that are known to colonize the skin, such as *Staphylococcus epidermidis* [2]. Physical and functional differences in the immature skin of preterm infants may alter the resident microbiota relative to full-term infants [3]. Furthermore, there are major differences in the early-life exposures of preterm infants compared to full-term infants, including frequent treatment with antibiotics, use of invasive lines and tubes, limited skin-to-skin contact with parents, and prolonged hospitalization. Understanding the development of skin microbiota and its relationship to other body sites may be of particular importance in this vulnerable population.

The skin acts as a physical barrier and immunologic interface to the external world including the local microbiota. The resident microbiota and immune system provide competitive exclusion of would-be pathogens. Interactions between the infant’s developing immune system and the early-life microbiota stimulate immune development, maturation, and tolerance. In germ-free mice, resident skin T cells exhibit attenuated cytokine responses in response to inflammatory stimuli [4]. In conventional mice, microbial colonization of the skin during a limited infant developmental window leads to an influx of antigen-specific activated regulatory T cells into the skin and the development of tolerance [5]. Understanding community dynamics of the skin microbiota in early life may reveal strategies to protect against infections and the development of later diseases such as atopy [6].

The objective of our study was to characterize the skin microbiota of preterm and full-term infants during their birth hospitalization. Second, we sought to determine the relationship of the skin microbiota with other body sites and the hospital environment. We hypothesized that skin microbial diversity would vary by gestational age, postnatal age, and body site, reflecting differences in environmental exposures and infant development. Further, we hypothesized that the skin microbiota of individual infants would share common features with other sites, suggesting an exchange of microbiota across body sites and the hospital environment during the establishment of microbial colonization.

## Methods

### Study cohort and sample collection

We enrolled preterm (< 37 weeks’ gestational age) and full-term (≥ 37 weeks’ gestational age) infants in the neonatal intensive care unit (NICU) or newborn nursery during their birth hospitalization. Infants in the newborn nursery roomed in with their mothers. The study was approved by the Duke Institutional Review Board (Pro00045553), and written informed consent was obtained from parents. A single set of samples was obtained from each infant at the time of study enrollment. Sterile swabs were used to collect the samples in a consistent manner from three body sites: the forehead and posterior auricular scalp (skin upper body; *n* = 108); the periumbilical region, inguinal folds, and upper thighs (skin lower body; *n* = 110); and the oral cavity (*n* = 123). Stool samples were only collected if a fresh specimen was available at the time of sampling (*n* = 38). In a subset of the preterm and full-term infants (*n* = 61), an additional sample was collected from the infant’s immediate environment. For these samples, a swab was rolled across commonly touched objects and immediately adjacent surfaces in the infant’s surroundings, including the bassinet or isolette (approximately 5 cm^2^ area of internal surface and handles), the temperature probe, and vital sign monitor (approximately 2 cm^2^ area). All samples were stored at − 80 °C until further processing.

### Sample processing

Genomic DNA was extracted from swabs using bead-beating and commercial extraction kits (Zymo Research). We performed PCR to amplify the V4 region of the 16S rRNA gene with barcode-indexed 515F-806R primers using previously described methods [7]. PCR reagents were pretreated with heat-labile shrimp DNase to remove contaminating double-stranded DNA. The DNase was inactivated by heating at 65 °C for 10 min before adding the genomic DNA template. PCR products were visualized on a 1.5% agarose gel, and biologic and environmental samples without a visible gel band of the expected size were removed from further processing. Amplicons were pooled in equimolar concentrations, purified by gel extraction, and sequenced on the Illumina MiSeq platform in two pools. Extraction controls were processed in the same manner as samples and included in the sequencing run to control for potential sources of DNA contamination in the extraction kits or buffers.

### Sequence processing

We used the QIIME platform to demultiplex, filter, and merge paired ends of the sequences [8]. Sequences sharing greater than 97% similarity were clustered into operational taxonomic units (OTUs). Taxonomy assignments were made by aligning representative sequences for each OTU to the SILVA bacterial database. The distribution of reads, OTUs, and genera per sample by sample type is presented in Additional file 1: Table S1. We removed samples with < 100 reads and sparse OTUs that did not have counts of more than 10 in at least 10% of samples. We also removed OTUs with > 1% abundance in the extraction control samples from the analysis, as these OTUs were likely to originate from sample preparation and reagents rather than the study infants (Additional file 2: Figure S1). However, we retained one *Staphylococcus* OTU that was present in the extraction controls (3% abundance), but was also the dominant *Staphylococcus* OTU found in the biological samples. We reasoned that laboratory contamination was unlikely to be the predominant source of the *Staphylococcus* OTU in the biological samples, given that the OTU accounted for a greater relative abundance of the microbiota within many biological sites than the extraction controls, and in an inverse ratio with other dominant contaminant OTUs. For example, the *Staphylococcus* OTU accounted for > 10% of total OTU abundance at the skin upper body site, while the most abundant OTU in the extraction control samples (genus: *Caldinitratiruptor*; 17% abundance in extraction controls) accounted for < 1% of total OTU abundance among the infant skin upper body samples. A median of 283 reads was removed as contaminants per infant sample. Sequence counts were normalized using cumulative sums scaling in the metagenomeSeq package [9].

### Statistical analysis

Infant characteristics were described for preterm and full-term infants. Wilcoxon rank-sum tests were used for comparison of continuous variables, and chi-square tests were used for categorical variables. Analysis and visualization of the microbial sequencing data were performed using R statistical software (version 3.2.2). Alpha diversity and beta diversity measures were examined using functions within the Phyloseq package [10]. We used adonis permutational multivariate analysis of variance (PERMANOVA) of generalized UniFrac (alpha = 0.5) and Bray-Curtis distances with 999 permutations to compare the microbiota community structure across body sites, with and without individual subjects included as a nested variable [11]. We applied PERMANOVA to evaluate the association between the skin microbiota and clinical characteristics, including gestational age (preterm vs. full term), postnatal age (< 3 vs. ≥ 3 days), diet (mostly human milk, mostly formula, or no feeds at the time of sampling), delivery mode (vaginal vs. cesarean delivery), and antibiotic use (any previous exposure), nested by sequencing run. Differences in relative abundance and the presence or absence of bacterial taxa between sample sites and gestational age groups were determined using the zero-inflated log-normal mixture model (fitFeatureModel) in metagenomeSeq [9]. These comparisons were made at the level of bacterial genus or the lowest taxonomic classification for OTUs that could not be assigned at the genus level.

To investigate the relationship between body sites and the environment, we used a Bayesian microbial source-tracking model to estimate the proportion of microbiota within each site that originated from the other sites [12]. The model was first applied to intraindividual site-source pairs among complete cases (i.e., infants with no missing data), then repeated with all subjects included in the model to examine interindividual site-source relationships.

## Results

### Study cohort

Samples were collected from a total of 129 infants, including 89 full-term infants and 40 preterm infants (Table 1). Seventy-seven of the 89 full-term infants (87%) were healthy infants who roomed in with their mothers during their birth hospitalization. The primary diagnoses for the 12 full-term infants who were admitted to the NICU are listed in Additional file 3: Table S2. A total of 85 (66%) of the infants were sampled in the immediate postnatal period (< 3 days of age), while 44 (34%) were sampled at later time points. The median postnatal age at the time of sampling was greater among the preterm infants than the full-term infants (*p* < 0.001; Table 1). Twenty-four (60%) of the preterm infants weighed less than 1000 g at birth (extremely low birth weight), and 9 (23%) infants weighed 1000–1500 g at birth (very low birth weight). Compared to the full-term infants, the premature infants were more likely to be multiples (twins or triplets; *p* < 0.001), to have been born by cesarean section (*p* < 0.001), and to have received antibiotics (*p* < 0.001; Table 1). Most of the premature infants (65%) were fed by a feeding tube (i.e., orogastric, nasogastric, or gastrostomy tube) at the time of sampling, with approximately half (48%) receiving mostly breast milk feeds. Major morbidities among the preterm infant cohort are presented in Additional file 3: Table S2. None of the infants had positive blood cultures during their hospitalization*.*

**Table 1:** Infant characteristics

	Preterm (*N* = 40)	Term (*N* = 89)
Baseline characteristics		
Birth weight (g), median (range)	845 (540–2508)	3365 (1820–4440)
Gestational age (weeks), median (range)	27 (23–36)	39 (37–42)
Female sex, *n* (%)	28 (70)	46 (52)
Multiple gestation, *n* (%)	13 (33)	7 (8)
Race, *n* (%)		
White	19 (48)	45 (51)
Black or African American	20 (50)	28 (31)
Asian	0 (0)	2 (2)
Native Hawaiian or other Pacific Islander	0 (0)	1 (1)
Unknown or not reported	1 (3)	13 (15)
Hispanic or Latino, *n* (%)	0 (0)	5 (6)
Mother hospital days prior to delivery, median (range)	3.5 (0–15)	0 (0–6)
Labor prior to delivery, *n* (%)	21 (53)	62 (70)
Prolonged rupture of membranes > 18 h, *n* (%)	6 (17)	14 (17)
Cesarean section, *n* (%)	30 (86)	36 (43)
Clinical factors at time of sampling		
Age at sampling (d), median (range)	42 (1–252)	1 (0–122)
Location, *n* (%)		
Neonatal intensive care unit	40 (100)	12 (13)
Mother’s room	0 (0)	77 (87)
Type of bed at time of sampling, *n* (%)		
Open crib	18 (45)	87 (98)
Warmer bed	1 (3)	2 (2)
Isolette	21 (53)	0 (0)
Diet, *n* (%)		
Mostly breast milk	19 (48)	56 (63)
Mostly formula	15 (38)	33 (37)
Any receipt of breast milk	32 (80)	63 (71)
No feeds prior to sampling	6 (15)	0 (0)
Primary feeding route, *n* (%)		
Breastfeeding	1 (3)	50 (56)
Bottle	7 (18)	38 (43)
Feeding tube	26 (65)	1 (1)
No feeds prior to sampling	6 (15)	0 (0)
Previous antibiotic exposure, *n* (%)	37 (93)	15 (17)

### Microbiota composition and diversity by site and gestational age

**Fig. 1:**
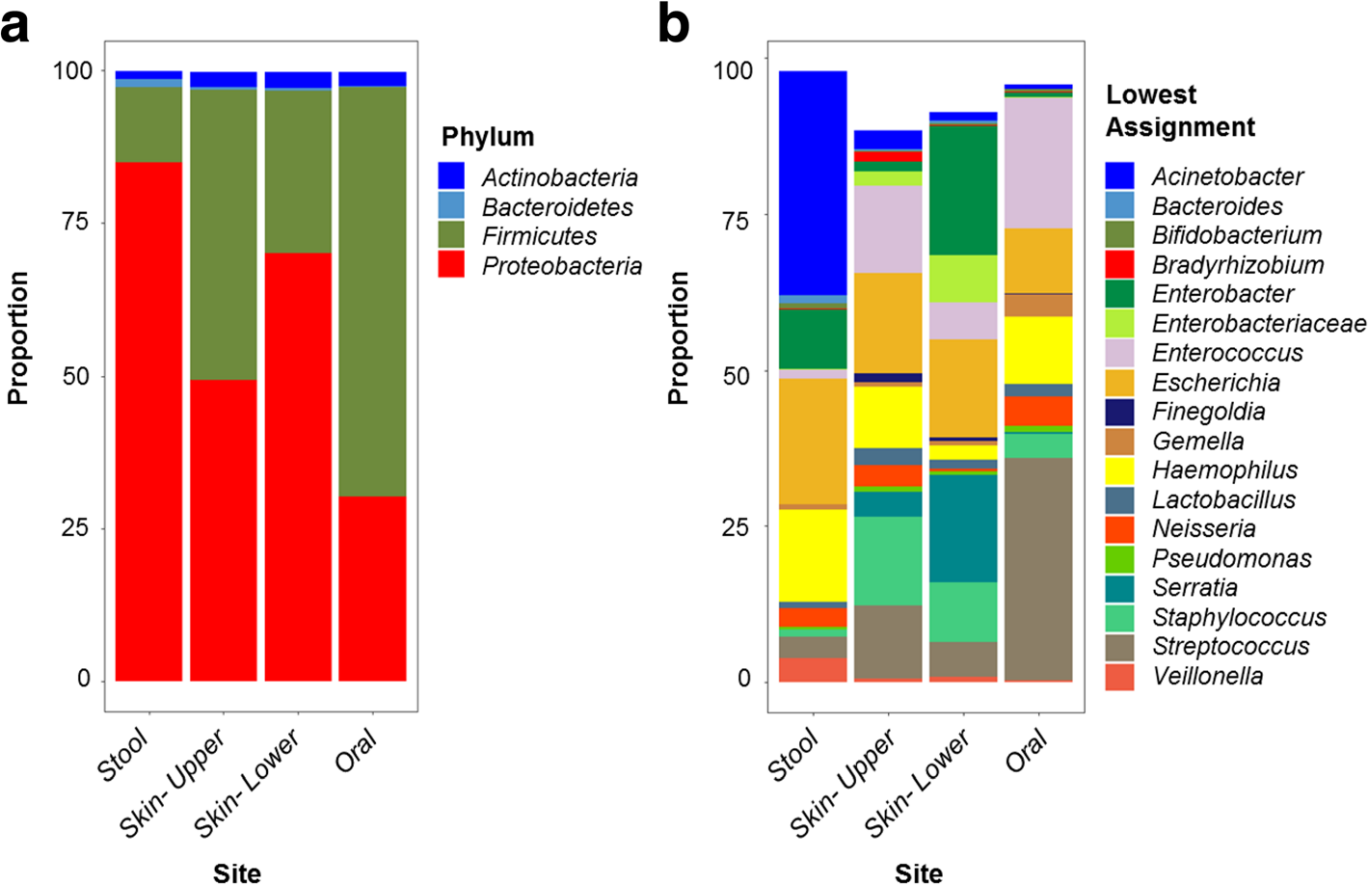
The relative abundance of bacterial genera at the level of phylum (**a**) and genus (**b**) for each body site. The lowest taxonomic classification is given for OTUs that were unable to be assigned a genus-level taxonomic classification

A total of 440 infant samples were analyzed. The median number of samples collected per subject was 4 (IQR 3–4). A total of 138 OTUs were included in the analysis following removal of sparse OTUs as well as 14 OTUs that were present in > 1% abundance in extraction controls (Additional file 2: Figure S1). The dominant bacterial phyla within each site were Proteobacteria and Firmicutes (Fig. 1a). At the genus level, taxa with the greatest relative abundance in the skin microbiota included *Staphylococcus*, *Streptococcus*, *Haemophilus*, *Enterococcus*, and multiple genera in the family *Enterobacteriaceae* including *Escherichia*, *Enterobacter*, and *Serratia* (Fig. 1b). *Streptococcus* was the most abundant bacterial genus within the oral cavity. The dominant bacterial genera within the fecal samples included *Acinetobacter*, *Escherichia*, *Haemophilus*, and *Enterobacter*. Of note, the majority of these stool samples were collected in the first days of life [median (interquartile range) age 1 (0–2) day] and therefore represent meconium, which is known to have a distinct microbiota compared to infant feces collected at later time points [13].

**Fig. 2:**
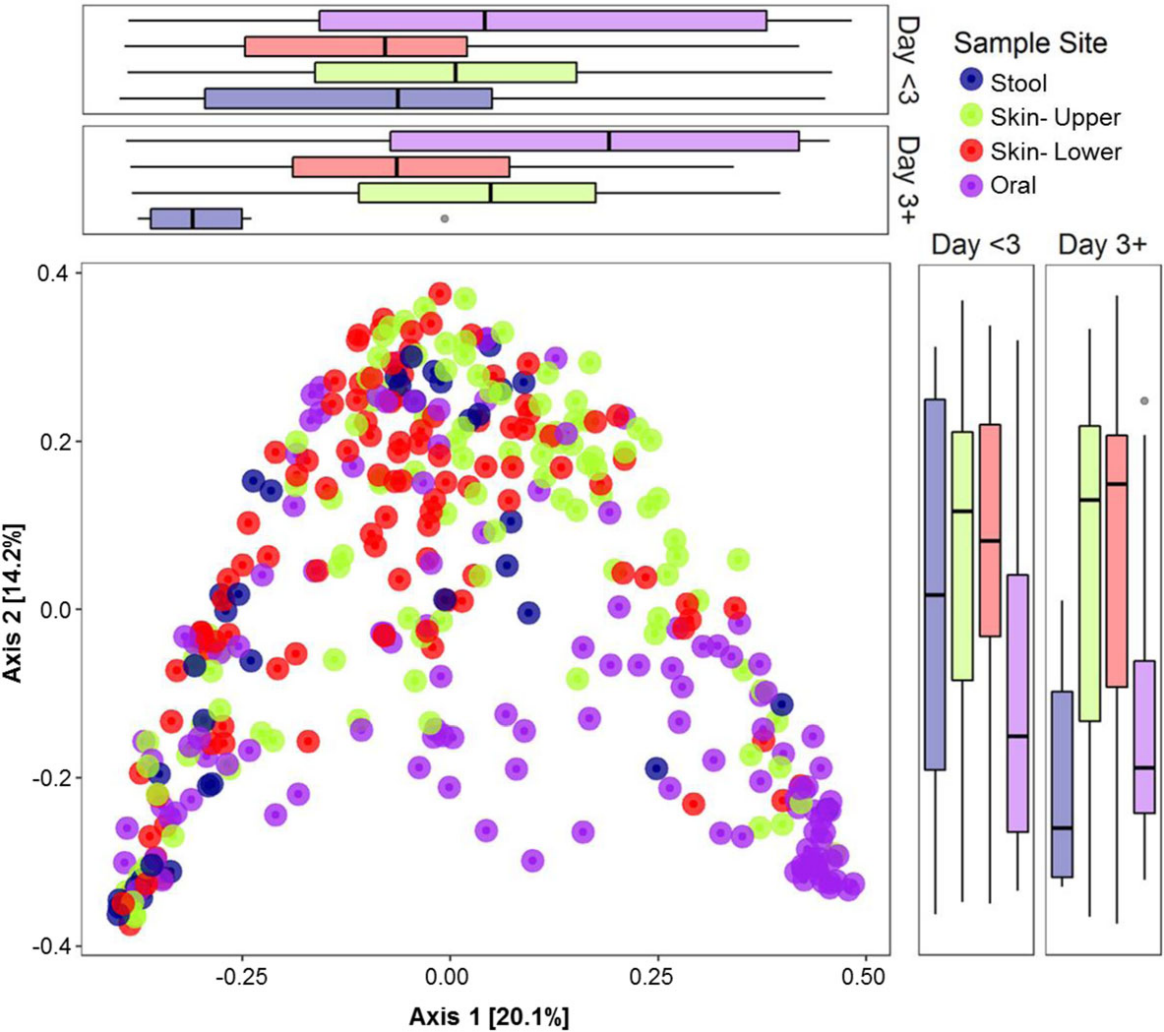
Principal coordinate analysis (PCoA) of generalized UniFrac distances. Each dot represents a sample and each color indicates a body site: stool (blue), skin upper body (green), skin lower body (red), and oral cavity (purple). The distribution of samples by body site is shown along the first and second axes of the PCoA plot. Along the first axis (PC1), the sample distribution differed significantly between the stool and skin upper body (*p* = 0.0048), the stool and oral cavity (*p* < 0.0001), skin upper body and skin lower body (*p* = 0.0049), skin upper body and oral cavity (*p =* 0.0049), and skin-lower body and oral cavity (*p <* 0.0001), but not between the stool and skin lower body (*p* = 0.1842; *p* values determined by pairwise Wilcoxon rank sum tests with Benjamini-Hochberg correction). Along the second axis (PC2), the oral samples differed from the skin upper body (*p <* 0.0001) and skin lower body (*p <* 0.0001), but other sites were not significantly different

We used principal coordinate analysis (PCoA) of generalized UniFrac distances to examine the relationship of microbial communities across body sites (Fig. 2) [11]. Microbiota community structure differed by site (adonis PERMANOVA *R*^2^ = 0.049; *n* = 379 samples), both when comparing sites across all subjects (*p* = 0.001) as well as when comparing sites nested within individual subjects (*p* = 0.001). However, there was a high degree of variation between samples without distinct spatial separation by body site. Differentiation between sites was greater among samples collected after the immediate postnatal period (i.e., postnatal age ≥ 3 days; Fig. 2). We observed similar relationships between sites using the non-phylogenetic Bray-Curtis distance metric (adonis *R*^2^ = 0.043, *p =* 0.001; Additional file 4: Figure S2A-B). Given that removal of contaminant OTUs may alter community composition, we also examined the relationship between body sites including the contaminant OTUs in the analysis. Here again, we found that body site accounted for a minor proportion of the variation between samples (*R*^2^ = 0.056, *p* = 0.001; Additional file 4: Figure S2C-D).

The community structure of the skin microbiota (*n* = 218 samples) differed by gestational age (adonis *R*^2^ = 0.016, *p* = 0.018; generalized UniFrac distances) and postnatal age (*R*^2^ = 0.016, *p* = 0.024), but not by antibiotic exposure (*p* = 0.211), diet (*p* = 0.305), or delivery mode (*p* = 0.089). Much of the variation in β-diversity between samples was not attributable to any of the measured clinical covariates, and between-site variation was notable within many of the individual subjects as well as twin pairs (Additional file 5: Figure S3).

Next, we used zero-inflated log-normal mixture models to identify discriminatory bacterial genera between the skin and other body sites among all infants, regardless of gestational age category. The skin upper body and lower body sites differed only in the relative abundance of *Streptococcus*, which was present in greater abundance in the upper body site (Additional file 6: Table S3). The oral cavity had a significantly higher abundance of *Streptococcus*, *Rothia*, and *Gemella* than both the skin upper and lower body sites, and greater abundance of *Neisseria* and *Haemophilus* than the skin lower body. The stool contained greater enrichment with *Aeromonas*, *Enterobacter*, *Enterobacteriaceae* (genus not classified), and an uncultured bacterium of the class γ-Proteobacteria than the skin upper body. In comparisons based on the presence or absence of bacterial taxa, *Corynebacteriaceae* was less likely to be present in the stool than the skin upper body (OR 0.16, 95% CI 0.03–0.56, *p*_adj_ = 0.046). There were no taxa with significant differences in relative abundance between the stool and the skin lower body site. After the immediate postnatal period (≥ 3 days old; *n* = 77 samples), the skin contained a greater relative abundance of *Staphylococcus* (*p*_adj_ < 0.001), *Veillonella* (*p*_adj_ = 0.038), *Finegoldia* (*p*_adj_ = 0.007), and lower abundance of *Neisseria* (*p*_adj_ = 0.038) and *Enterobacter* (*p*_adj_ = 0.017) than in the first days of life (*n* = 141 samples).

**Fig. 3:**
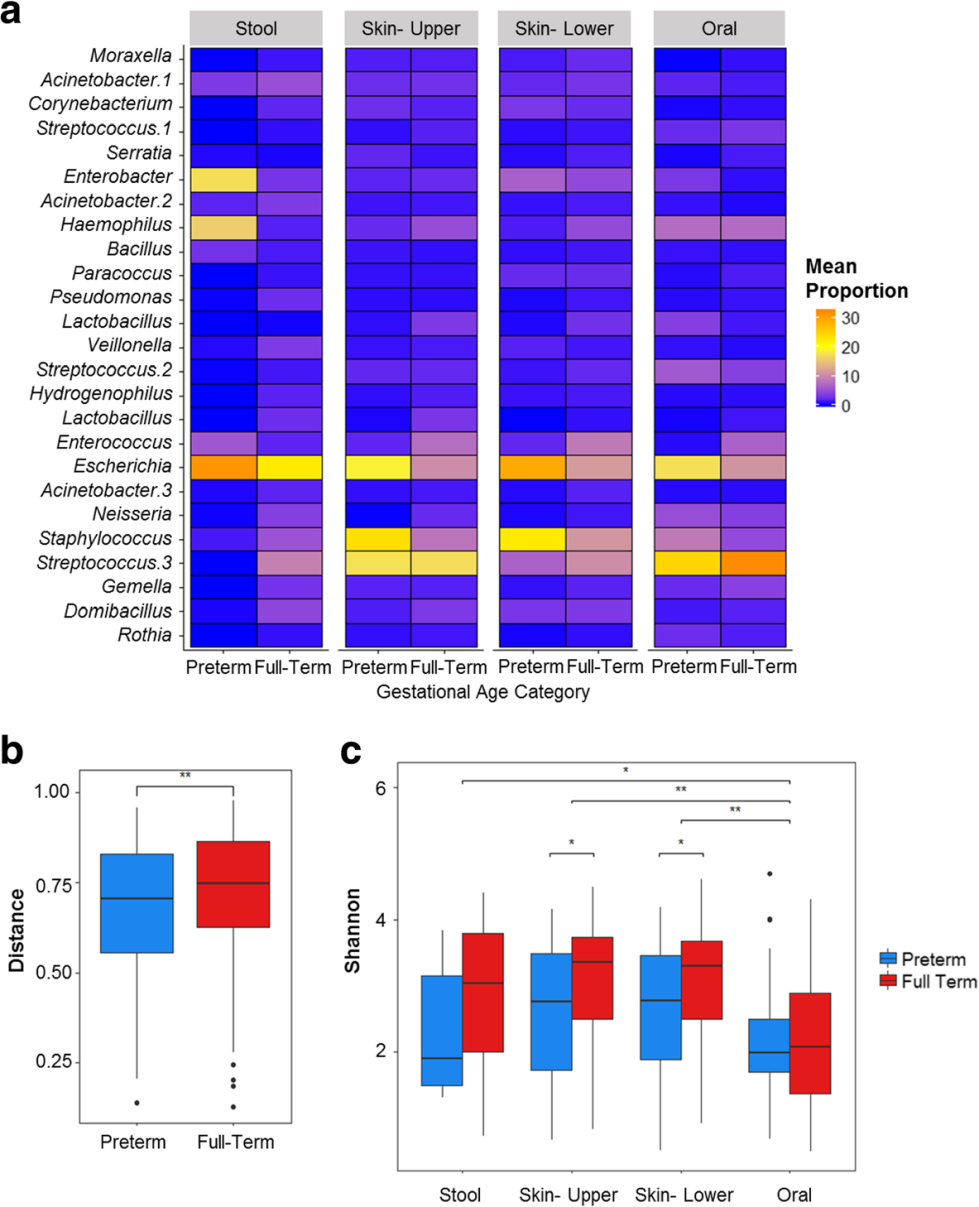
Comparison of the preterm and full-term infant microbiota across body sites. **a** The mean proportion (per sample) of the top OTUs within each body site in preterm and full-term infants. **b** Intra-individual generalized UniFrac distances between body sites in preterm and full-term infants. Between-site distances were greater in full-term infants than preterm infants (median 0.75 vs 0.70, *p* = 0.006). **c** Shannon diversity across body sites and gestational age groups. Alpha diversity was significantly lower among oral samples than the stool, skin upper body, and skin lower body (*p* values determined by pairwise Wilcoxon rank sum tests with Benjamini-Hochberg correction). Shannon diversity did not differ significantly across the other body sites. Alpha diversity was lower in the skin among preterm infants compared to the full-term infants. *p < 0.05, **p < 0.01

We examined differences in the microbiota between preterm infants and full-term infants (Fig. 3). The skin microbiota of full-term infants (*n* = 147 samples) contained a greater relative abundance of *Neisseria*, while the preterm infants (*n* = 71 samples) had a greater abundance of *Staphylococcus*, *Bacillus*, *Escherichia*, *Enterobacter*, and other taxa within the Gammaproteobacteria class (Additional file 7: Table S4). The skin microbiota of preterm infants was also more likely to contain bacteria within the *Stenotrophomonas* genus (OR 2.60; 95% CI 1.28–5.32; *p*_adj_ = 0.037). The oral cavity of preterm infants (*n* = 39 samples) had a greater abundance of *Stenotrophomonas*, *Lactococcus*, and *Enterobacter* than the full-term infants (*n* = 84 samples). Within individual subjects, between-site generalized UniFrac distances were significantly higher in the full-term infants than the preterm infants (Fig. 3b) [11].

We compared alpha diversity, as measured by the Shannon diversity index, between body sites (Fig. 3c). Alpha diversity was significantly lower among oral samples than the stool (*p* = 0.014), skin upper body (*p* < 0.0001), and skin lower body (*p <* 0.0001; Fig. 3c). Both skin sites (upper and lower bodies) had significantly lower alpha diversity among preterm infants than full-term infants (*p* = 0.030 and *p* = 0.017, respectively). Alpha diversity within the stool and oral cavity were not significantly different between gestational age groups. The diversity of the fecal microbiota was lower among samples collected at postnatal age > 2 days than samples collected in the first days of life (median 1.89 vs. 3.12, *p* = 0.037), but there were no significant differences in diversity by postnatal age within the skin or oral microbiota. Shannon indices did not differ significantly by delivery mode (C-section vs. vaginal delivery) at any of the sites (data not shown).

### Microbiota in the hospital environment

Environmental samples were obtained in a subset of the infants (20 preterm infants and 41 full-term infants) to determine the relationship between each infant’s skin microbiota with the hospital environment. All of the preterm infants and 6 (15%) of the full-term infants were located in the NICU at the time of sampling; the remaining 35 (85%) full-term infants were located in their mothers’ rooms. The environment was enriched in many of the same genera found in the infant skin and other body sites, including *Escherichia*, *Staphylococcus*, and *Streptococcus* (Additional file 8: Figure S4A). These taxa dominated the environmental samples from both preterm and full-term infants. However, the preterm infant environmental microbiota had a greater relative abundance of several taxa, including members of the Gammaproteobacteria class that were also more abundant in the skin microbiota of the preterm infant compared to the full-term infant (Additional file 7: Table S4). Taxa with greater relative abundance in the skin microbiota than the environment included *Enterococcus* (*p* = 0.034, skin upper; *p* = 0.003, skin lower), *Streptococcus* (*p* = 0.008, skin upper), *Bacteroides* (*p* = 0.039, skin upper; *p* = 0.026, skin lower), *Anaerobacillus* (*p* = 0.031, skin lower), and *Enterobacter* (*p =* 0.026, skin lower). Median generalized UniFrac distances between the infant microbiota and the environment were lower among preterm infants than full-term infants, but the difference was only statistically significant for the stool samples (Additional file 8: Figure S4B).

### Relationship between body sites and the hospital environment

**Fig. 4:**
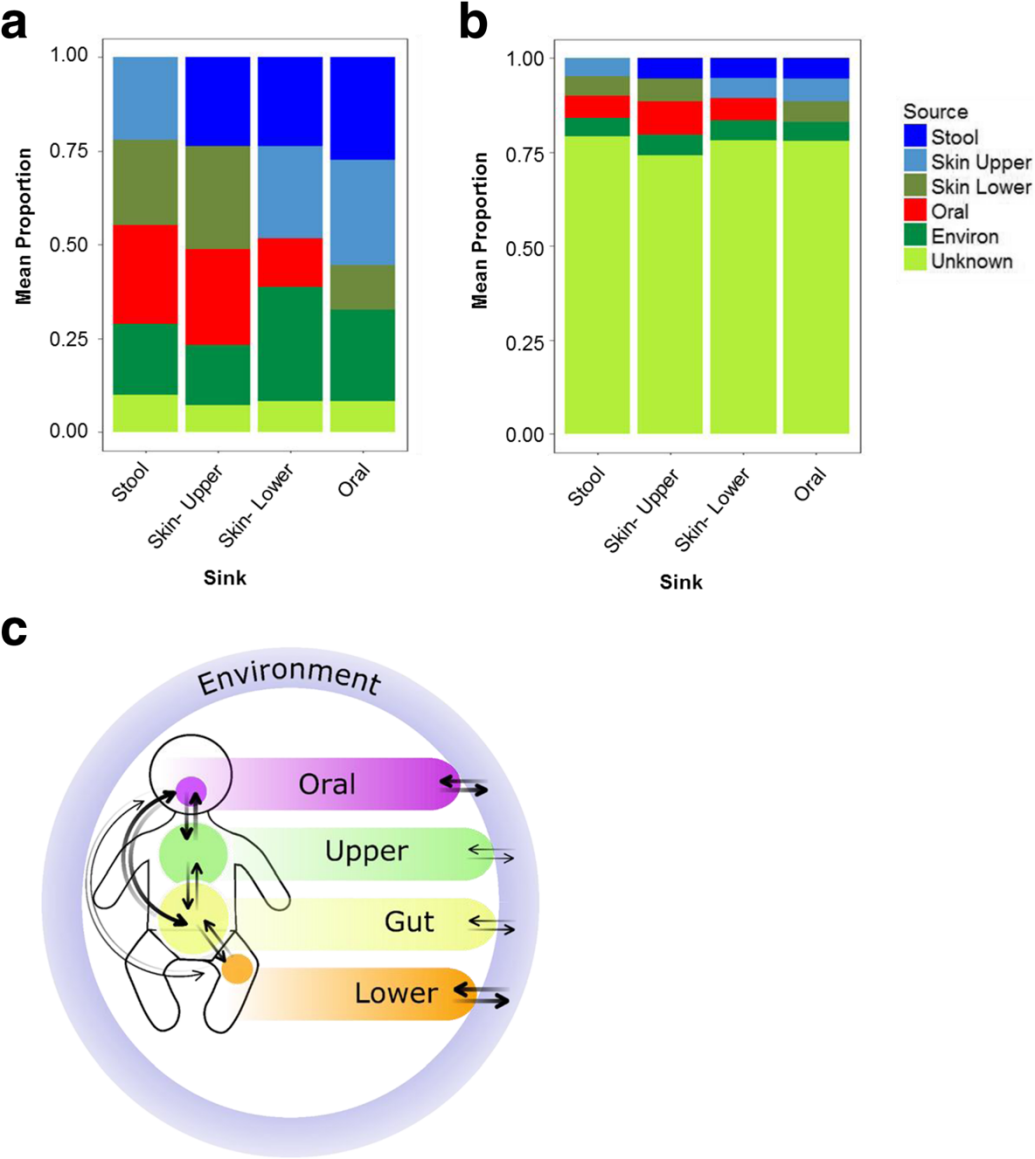
Source tracking of microbiota across body sites. The mean proportion of microbiota within each site (“sink”) attributable to each source are shown among intraindividual (**a**) and interindividual (**b**) sink-source pairs. Intraindividual relationships between sites are further depicted (**c**), with the weight of arrows between sites showing the relative contribution of each source

We used bacterial source tracking to explore the predicted sources of microbiota within each body site (Fig. 4). First, we applied the source-tracking model using only intra-individual site-source pairs. The predicted sources of microbiota for each site varied between individual subjects, but the majority of the microbiota within each site was attributable to the infant’s other body sites (Fig. 4a). The skin microbiota appeared to both receive microbiota from and contribute to the microbiota of other body sites (Fig. 4a, c). The hospital environment was the predicted source for approximately one quarter of the microbiota at each body site. The source-tracker model was then reapplied to investigate site-source relationships between infants. In this model, the vast majority of the microbiota within each site could not be attributed to a known source (Fig. 4b). These findings suggest that the infant microbiota is more closely related to their own environment and other body sites than to the microbiota of other infants.

## Discussion

In this study, we characterized the skin microbiota of hospitalized preterm and full-term infants and described its relationship to other body sites and the hospital environment. The skin microbiota varied between individuals and by gestational age, postnatal age, and body region. It was enriched in typical skin-associated bacteria such as *Streptococcus* and *Staphylococcus* but also in many taxa that are typically associated with the gut microbiome, including *Escherichia*, *Enterobacter*, and *Enterococcus*. Many of the abundant taxa in the skin microbiota are common causes of late-onset sepsis in preterm infants [2]. While many of same genera were present in skin upper body and skin lower body sites, the relative abundance of bacteria within the Proteobacteria phylum was higher in the skin lower body site, potentially reflecting fecal contamination of the skin. The high abundance of potential pathogens in this skin region is worth noting given the frequent use of umbilical and femoral central vascular catheters in critically ill infants. Our bacterial source-tracking model indicated that the skin microbiota both acquires and contributes microbiota to other body sites, suggesting that body sites can serve as bacterial reservoirs for one another in infancy.

Studies in adults have shown that the microbiota is highly differentiated in structure and function across body sites and skin surfaces [14, 15]. In our study, we found differences in microbiota community structure, alpha diversity, and relative abundances of bacterial taxa between sites. In general, however, discrimination between body sites was relatively weak. Similar findings have been reported in other preterm and full-term neonatal cohorts, with greater distinction between sites occurring in early infancy [16–19]. We found that site differentiation was greater for samples collected after the first two postnatal days, suggesting the rapid development of niche selection. The dynamic progression from nonspecific colonization with a common inoculum to the formation of site-specific microbial communities is likely driven, in part, by concurrent physical, chemical, and immunologic changes in the neonatal period and early infancy. A recent study illustrated that in two preterm infants, identical strains colonized the infant’s oral cavity, skin, and gut, but demonstrated differential growth rates by site [20].

The extent to which environmental acquisition of microbes contributes to the development of skin microbial communities is not entirely understood. A recent study demonstrated that the skin microbiota differs between children living in rural and urban environments, particularly during early childhood (ages 1–4), suggesting that the living environment alters the development of the skin microbiota [21]. The open landscape and pro-tolerogenic immune bias of the neonate may make the skin more susceptible to invasion by environmental microbes than in later life, but the timing of this window of susceptibility and the specific host and microbial community factors that confer colonization resistance to environmental microbes are unclear. In adults, skin microbial communities are largely stable over time and microbial community niches appear to be maintained primarily by growth of indigenous strains rather than the acquisition of new strains from the environment [14]. In our study, we found substantial overlap between the infant’s skin, gut, and oral microbiota and the hospital environment. While we cannot fully determine the directionality of transfer of microbiota between the infant and the environment from our cross-sectional data, a substantial proportion of microbiota in the infant skin and other sites were attributed to the hospital environment in our source tracking model. In addition to the physical environment, infants acquire microbiota from their mothers through delivery, skin-to-skin contact, and breast milk feeding [22]. We did not collect samples from mothers and thus could not determine the relative contribution of maternal and environmental sources of microbiota in our study. Critically ill infants with prolonged hospital stays may acquire a greater proportion of their initial microbiota from the hospital environment than healthy infants, given their lack of physical contact with their mothers and the often delayed introduction of human milk feedings. Further study is needed to understand the acquisition and persistence of the environmental microbiota in these infants and its potential effects on subsequent maturation of the microbiome and clinical outcomes.

The influence of delivery mode on the neonatal microbiome has been a major area of interest. Several studies have suggested a difference in the microbiota of infants who are born by vaginal delivery versus those born by C-section [18, 23–25]. A recent study by Chu et al. examined the influence of delivery mode on infant microbial communities in a cohort of infants with a mean gestational age of 38 ± 2.5 weeks [17]. They identified modest differences in the microbiota of infants by delivery mode among 157 infants sampled at the time of birth, but there were no appreciable differences in microbiota community structure or diversity among 60 infants who had repeat sampling at age 4–6 weeks. Further, there were no notable differences in microbial community function by delivery mode in a subset of infants who were studied by whole-genome shotgun sequencing. In the current study, mode of delivery did not appear to have a strong influence on the infant microbiota. We did not see significant differences in alpha or beta diversity between infants born by C-section and those born by vaginal delivery.

We found that the skin microbiota of preterm infants differed from that of the full-term infants, with greater enrichment of *Staphylococcus* and several taxa that are typically associated with the fecal microbiota, such as *Escherichia*. Despite sampling at later time points, UniFrac distances between sites were modestly lower in preterm infants than full-term infants, suggesting less site differentiation. Alpha diversity was also lower in the skin of preterm infants, which may render the skin microbiota more susceptible to invasion by pathogens. However, there was overlap between many of the full-term and preterm infant samples. The lack of strong differentiation between gestational age groups may indicate that the susceptibility of preterm infants to infection is primarily driven by differences in host biology, including immune function and barrier integrity, rather than by differences in skin colonization. Our data are limited in that none of the infants developed bloodstream infections in our cohort and we did not examine the full genetic potential of the microbiota. It is possible that the microbiota of preterm infants harbored more virulent bacterial strains than the full-term infants, despite sharing many of the same OTUs. A recently published study found no association between neonatal sepsis and the skin microbiome, but the study was limited by small sample size (*n* = 12) and inconsistent timing of sample collection between subjects relative to the onset of sepsis [26].

The study reported herein has limitations. The cross-sectional nature of our study design limited our ability to delineate maturational changes and interactions between body sites and the environment over time, and to explore the relationships between the skin microbiota and relevant clinical outcomes. The median postnatal age at the time of sampling was lower in the full-term infants than the preterm infants. Differences in the time of sampling combined with the cross-sectional study design may have confounded the comparisons between gestational age and postnatal age groups. We lacked the genetic resolution to be able to determine strain variation within taxa across sites and individuals. Further, we did not evaluate the functional capacity of the microbiota, limiting our ability to say whether the compositional and structural differences we observed corresponded to functional differences in microbial communities. Future longitudinal studies directed at elucidating the interactions between the infant microbiota and environmental sources through metagenomics may provide a more comprehensive understanding of microbiota assembly in infancy.

## Conclusions

In conclusion, the skin microbiota was highly variable across individuals in this large cohort of hospitalized full-term and preterm infants. The skin microbiota differed across stages of infant development, shared commonalities with the developing microbial communities at other body sites, and was predicted to be, in part, shaped by microbiota acquired from the hospital environment.

## Additional files

Additional file 1: **Table S1.** Raw sequencing reads, OTUs, and genera per sample by sample type. (DOCX 13 kb)

Additional file 2: **Figure S1.** Contaminant OTUs identified in extraction control samples. A. Relative abundance of bacterial taxa in extraction control samples. B. OTUs with greater than 1% relative abundance in extraction controls. These OTUs were excluded from subsequent analyses as they were presumed to be contaminants, except the highlighted *Staphylococcus* OTU that was found to be the dominant *Staphylococcus* OTU in the biological samples. C. Relative abundance of the contaminant OTUs (in aggregate) that were excluded from subsequent analyses within each sample site. The contaminant OTUs contributed to a minority of the total OTU abundance in each of the sample sites. OTU = operational taxonomic unit. (PPTX 264 kb)

Additional file 3: **Table S2.** Diagnoses and morbidities among infants admitted to the neonatal intensive care unit. (DOCX 12 kb)

Additional file 4: **Figure S2.** Principal coordinates analysis (PCoA) of samples across body sites. PCoA of infant samples excluding contaminant OTUs (A, B) or including contaminant OTUs (C, D). Similar relationships between body sites are seen using generalized UniFrac distances (A, C) and Bray-Curtis distances (B, D). In panel C, the first and second axes are rotated to keep the orientation of samples consistent with the other panels, but it should be noted that the vertical axis accounts for the majority of the variation between samples in this panel. (PPTX 717 kb)

Additional file 5: **Figure S3.** Skin and oral microbiota of twin pairs. A. Characteristics of the five twin pairs that were included in the study are shown, including the gestational age (preterm or full-term), the postnatal age (in days) at the time of sample collection, and the percentage of oral and skin OTUs that were shared between the infants in each twin pair. The relative abundance of the top bacterial genera within the skin and oral microbiomes are shown for the individual infants (Twin A, Twin B) within each twin pair (Twin Pairs 1–5). B. Principal coordinates analysis of samples from the twins based on generalized UniFrac distances. The twin pairs (1–5) are grouped by color. (PPTX 363 kb)

Additional file 6: **Table S3.** Bacterial taxa with differences in abundance between body sites. (DOCX 12 kb)

Additional file 7: **Table S4.** Bacterial taxa with differences in abundance between gestational age groups. (DOCX 13 kb)

Additional file 8: **Figure S4.** The environmental microbiota of preterm and full-term infants. A. Relative abundance of the top genera in the hospital environment. B. Generalized UniFrac distances between infant body sites and their corresponding environmental samples. Median distances were lower among preterm infants. *p < 0.05. (PPTX 118 kb)

## Abbreviations

NICU: Neonatal intensive care unit
OTU: Operational taxonomic unit
