# Supplementary material for: Early-life skin microbiota in hospitalized preterm and full-term infants
Source: Microbiome. 2018 May 31;6:98. doi: 10.1186/s40168-018-0486-4 (PMC5984431; doi:10.1186/s40168-018-0486-4)
Supplement: Supplementary file 1 — Table S1. Raw sequencing reads, OTUs, and genera per sample by sample type. (DOCX 13 kb) [file 40168_2018_486_MOESM1_ESM.docx]

**Table S1. Raw sequencing reads, OTUs, and genera per sample by sample type.**

| **Sample Type** | **Reads per sample, Median (IQR)** | **OTUs per sample,**  **Median (IQR)** | | | **Genera per sample,**  **Median (IQR)** | | |
| --- | --- | --- | --- | --- | --- | --- | --- |
|  |  | **OTUs with counts >1** | **OTUs with counts >10** | **OTUs with counts >100** | **Genera with counts >1** | **Genera with counts >10** | **Genera with counts >100** |
| **Skin- Upper** | 8829 (4743, 23771) | 162 (98, 276) | 60 (38, 106) | 14 (8, 21) | 49 (35, 68) | 37 (27, 51) | 13 (7, 19) |
| **Skin- Lower** | 8511 (3881, 19037) | 154 (96, 278) | 56 (35, 88) | 12 (6, 20) | 48 (37, 63) | 36 (24, 46) | 11 (7, 17) |
| **Oral** | 19857 (8609, 39706) | 182 (116, 323) | 63 (42, 106) | 10 (6, 18) | 40 (29, 60) | 23 (16, 37) | 7 (3, 12) |
| **Stool** | 30867 (5652, 57978) | 236 (146, 373) | 81 (52, 130) | 16 (9, 23) | 45 (29, 83) | 27 (16, 53) | 9 (5, 16) |
| **Environment** | 5123 (1955, 8399) | 139 (59, 188) | 46 (28, 69) | 9 (5, 15) | 51 (28, 68) | 32 (20, 47) | 9 (5, 16) |
