# Supplementary figures and images for: Early-life skin microbiota in hospitalized preterm and full-term infants
Source: Microbiome. 2018 May 31;6:98. doi: 10.1186/s40168-018-0486-4 (PMC5984431; doi:10.1186/s40168-018-0486-4)

## Slide 1
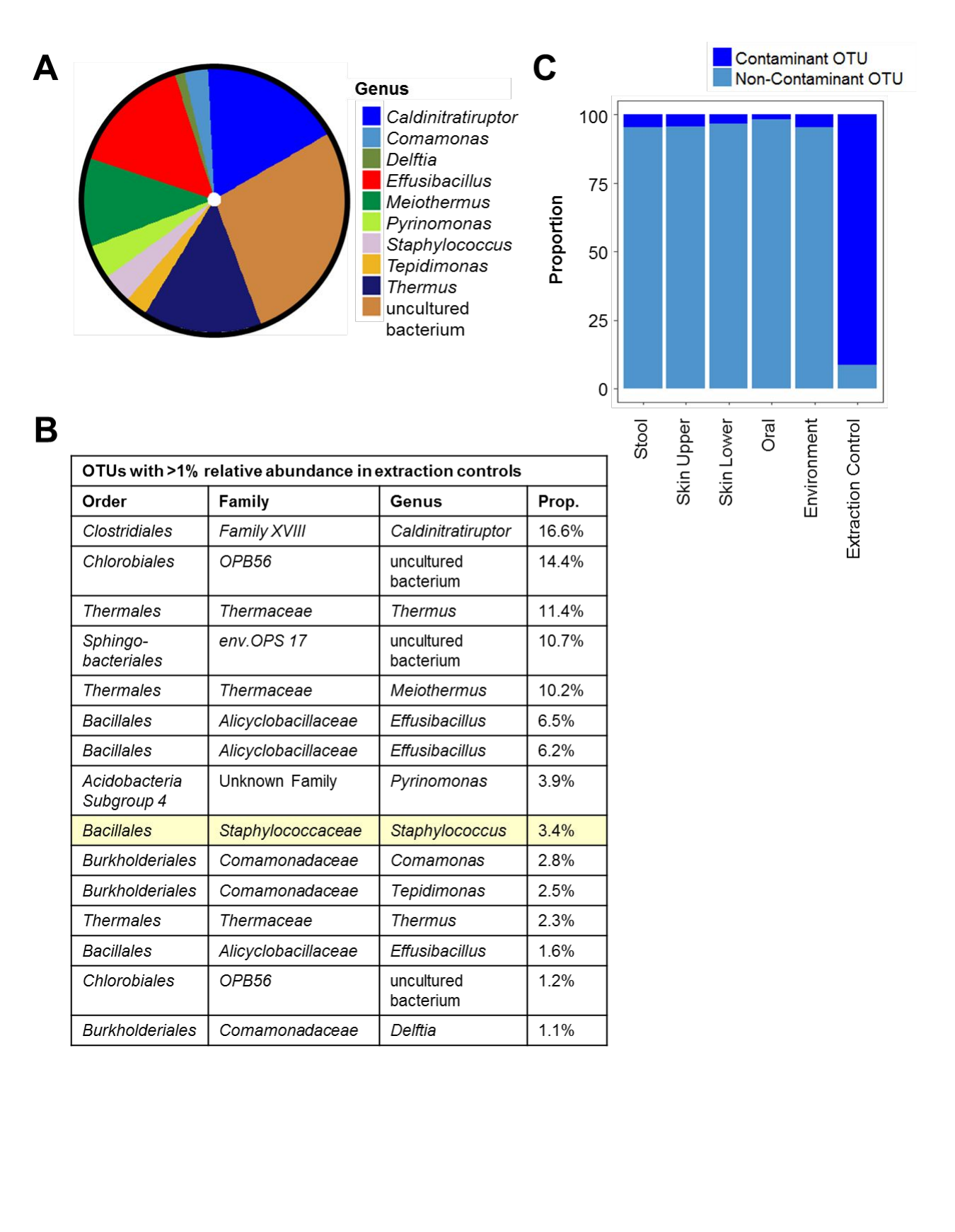

Supplement: Supplementary file 2 — Figure S1. Contaminant OTUs identified in extraction control samples. A. Relative abundance of bacterial taxa in extraction control samples. B. OTUs with greater than 1% relative abundance in extraction controls. These OTUs were excluded from subsequent analyses as they were presumed to be contaminants, except the highlighted Staphylococcus OTU that was found to be the dominant Staphylococcus OTU in the biological samples. C. Relative abundance of the contaminant OTUs (in aggregate) that were excluded from subsequent analyses within each sample site. The contaminant OTUs contributed to a minority of the total OTU abundance in each of the sample sites. OTU = operational taxonomic unit. (PPTX 264 kb) [file 40168_2018_486_MOESM2_ESM.pptx]

## Slide 1
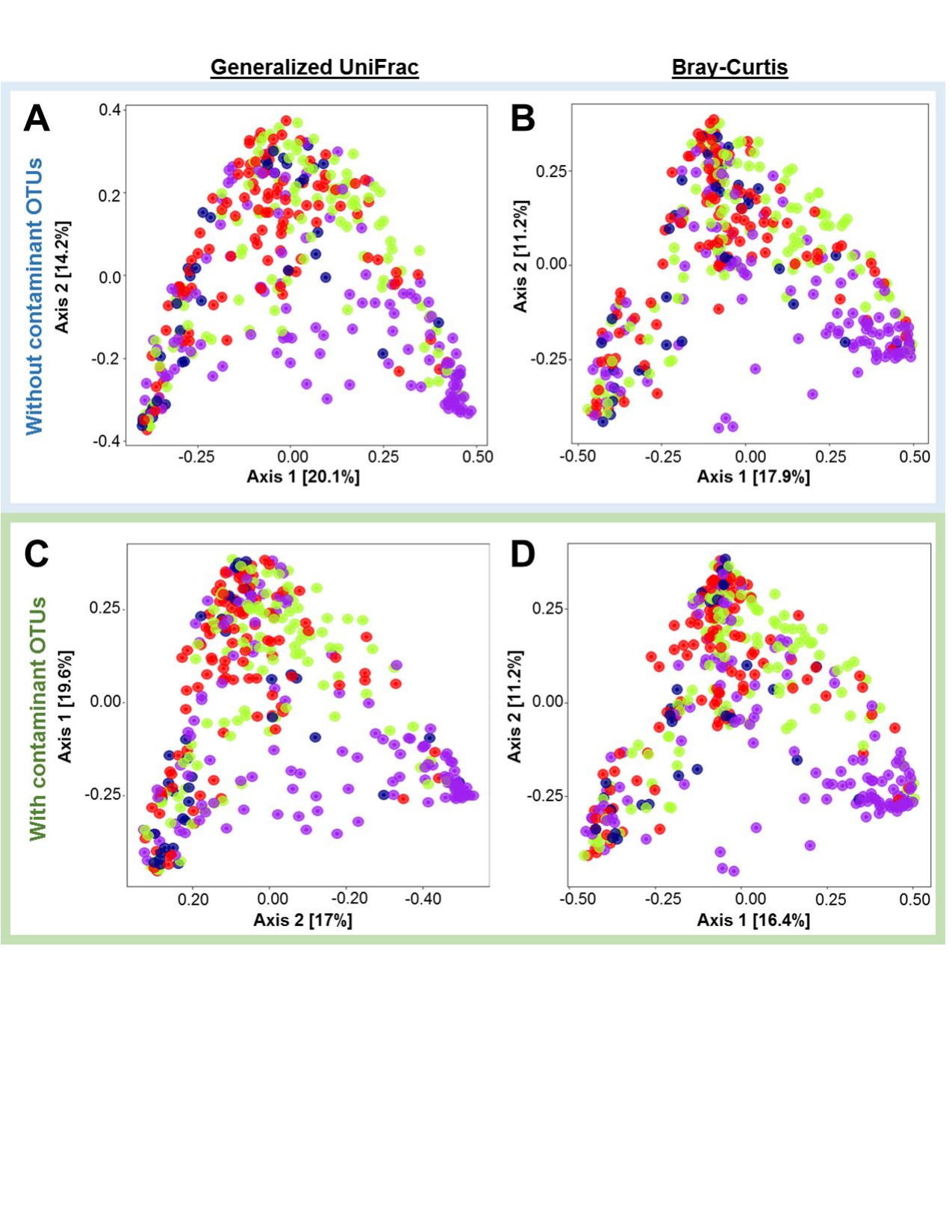

Supplement: Supplementary file 4 — Figure S2. Principal coordinates analysis (PCoA) of samples across body sites. PCoA of infant samples excluding contaminant OTUs (A, B) or including contaminant OTUs (C, D). Similar relationships between body sites are seen using generalized UniFrac distances (A, C) and Bray-Curtis distances (B, D). In panel C, the first and second axes are rotated to keep the orientation of samples consistent with the other panels, but it should be noted that the vertical axis accounts for the majority of the variation between samples in this panel. (PPTX 717 kb) [file 40168_2018_486_MOESM4_ESM.pptx]

## Slide 1
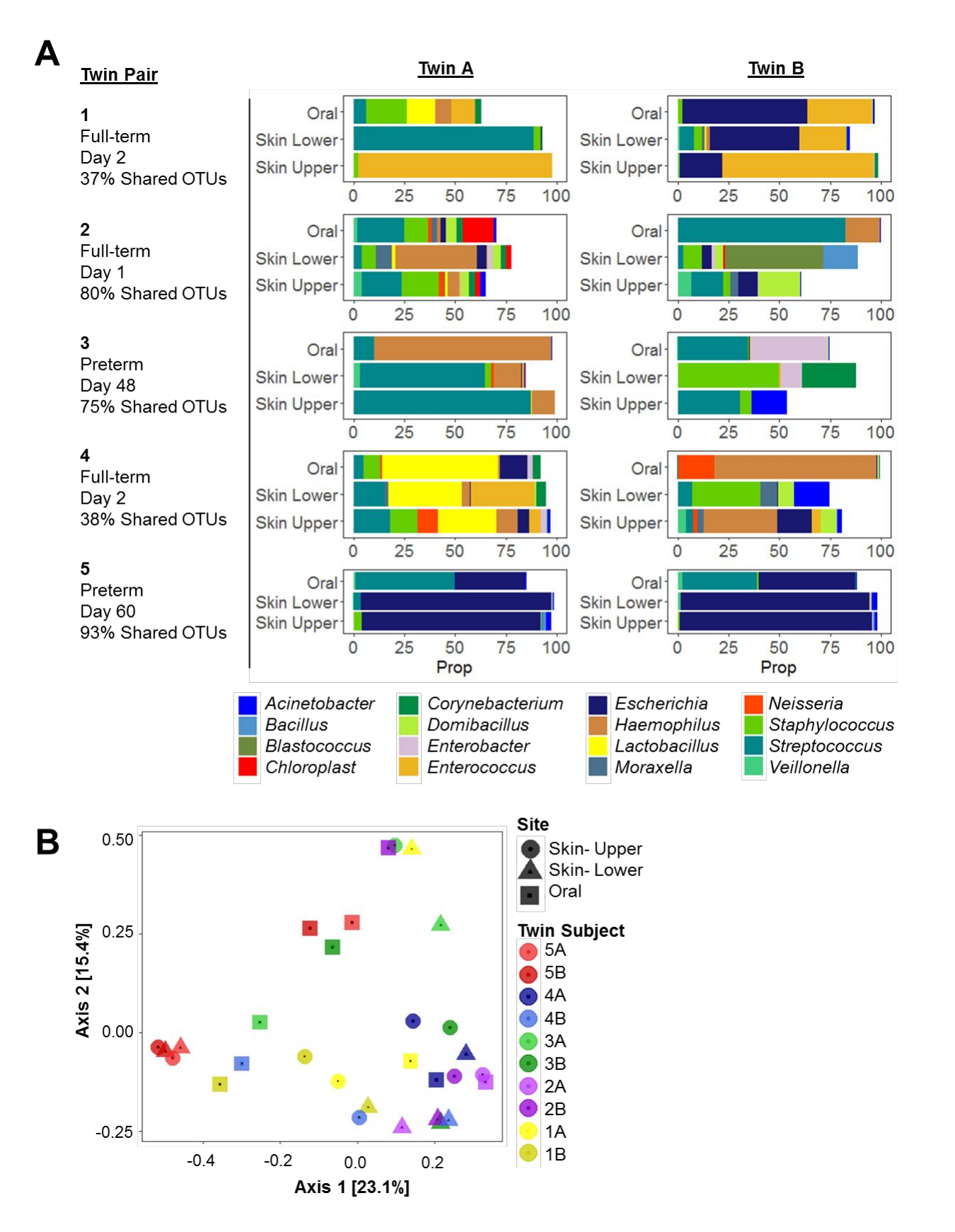

Supplement: Supplementary file 5 — Figure S3. Skin and oral microbiota of twin pairs. A. Characteristics of the five twin pairs that were included in the study are shown, including the gestational age (preterm or full-term), the postnatal age (in days) at the time of sample collection, and the percentage of oral and skin OTUs that were shared between the infants in each twin pair. The relative abundance of the top bacterial genera within the skin and oral microbiomes are shown for the individual infants (Twin A, Twin B) within each twin pair (Twin Pairs 1–5). B. Principal coordinates analysis of samples from the twins based on generalized UniFrac distances. The twin pairs (1–5) are grouped by color. (PPTX 363 kb) [file 40168_2018_486_MOESM5_ESM.pptx]

## Slide 1
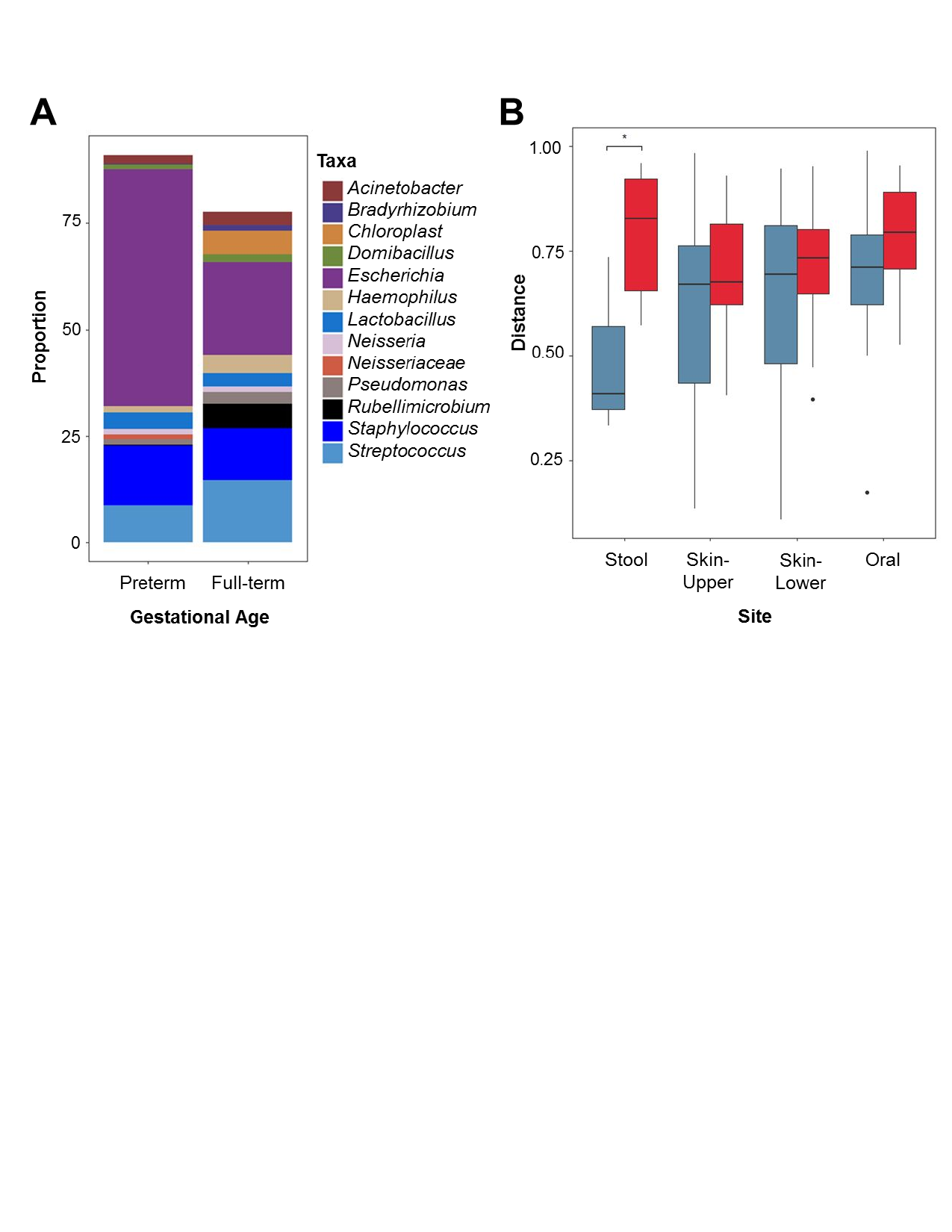

Supplement: Supplementary file 8 — Figure S4. The environmental microbiota of preterm and full-term infants. A. Relative abundance of the top genera in the hospital environment. B. Generalized UniFrac distances between infant body sites and their corresponding environmental samples. Median distances were lower among preterm infants. *p < 0.05. (PPTX 118 kb) [file 40168_2018_486_MOESM8_ESM.pptx]
