# Supplementary material for: Early-life skin microbiota in hospitalized preterm and full-term infants
Source: Microbiome. 2018 May 31;6:98. doi: 10.1186/s40168-018-0486-4 (PMC5984431; doi:10.1186/s40168-018-0486-4)
Supplement: Supplementary file 3 — Table S2. Diagnoses and morbidities among infants admitted to the neonatal intensive care unit. (DOCX 12 kb) [file 40168_2018_486_MOESM3_ESM.docx]

**Table S2. Diagnoses and morbidities among infants admitted to the neonatal intensive care unit.**

| **Full term infants (N=12), admitting diagnoses, n (%)** |  |
| --- | --- |
| Feeding problems  Neonatal abstinence syndrome  Respiratory problems  Small for gestational age  Gastroschisis  Hypoxic-ischemic encephalopathy  Seizures  Fever  Hypoglycemia | 2 (17)  2 (17)  2 (17)  1 (8)  1 (8)  1 (8)  1 (8)  1 (8)  1 (8) |
| **Preterm infants (N=40), major morbidities, n (%)** |  |
| Necrotizing enterocolitis or spontaneous intestinal perforation  Ligation of a patent ductus arteriosus  Severe intraventricular hemorrhage  Chronic lung disease  Severe retinopathy of prematurity  Culture-proven sepsis | 3 (8)  4 (10)  0 (0)  13 (33)  5 (13)  0 (0) |
