# Supplementary material for: Early-life skin microbiota in hospitalized preterm and full-term infants
Source: Microbiome. 2018 May 31;6:98. doi: 10.1186/s40168-018-0486-4 (PMC5984431; doi:10.1186/s40168-018-0486-4)
Supplement: Supplementary file 6 — Table S3. Bacterial taxa with differences in abundance between body sites. (DOCX 12 kb) [file 40168_2018_486_MOESM6_ESM.docx]

**Table S3. Bacterial taxa with differences in abundance between body sites**

| **Sites and Taxa** | **Log fold change** | **P** | **Adjusted P*** |
| --- | --- | --- | --- |
| **Skin Upper > Skin Lower** |  |  |  |
| *Streptococcus* | 1.05 | 0.0005 | 0.0316 |
| **Oral > Skin Upper** |  |  |  |
| *Rothia* | 1.036 | 0.0003 | 0.0062 |
| *Streptococcus* | 0.895 | <0.0001 | 0.0006 |
| *Gemella* | 0.875 | 0.0002 | 0.0062 |
| **Oral > Skin Lower** |  |  |  |
| *Rothia* | 3.264 | <0.0001 | <0.0001 |
| *Streptococcus* | 3.012 | <0.0001 | <0.0001 |
| *Gemella* | 2.462 | <0.0001 | 0.0011 |
| *Neisseria* | 2.333 | 0.0002 | 0.0027 |
| *Haemophilus* | 1.487 | 0.0025 | 0.0255 |
| **Stool > Skin Upper** |  |  |  |
| *Aeromonas* | 2.284 | 0.0002 | 0.0139 |
| Uncultured bacterium** | 2.160 | 0.0033 | 0.0491 |
| *Enterobacteriaceae* | 1.609 | 0.0020 | 0.0393 |
| *Enterobacter* | 1.526 | 0.0014 | 0.0393 |

*False Discovery Rate (FDR) adjusted

**Class-*Gammaproteobacteria*, Order-*B38*, Family-uncultured bacterium
