# Supplementary material for: Early-life skin microbiota in hospitalized preterm and full-term infants
Source: Microbiome. 2018 May 31;6:98. doi: 10.1186/s40168-018-0486-4 (PMC5984431; doi:10.1186/s40168-018-0486-4)
Supplement: Supplementary file 7 — Table S4. Bacterial taxa with differences in abundance between gestational age groups. (DOCX 13 kb) [file 40168_2018_486_MOESM7_ESM.docx]

**Table S4. Bacterial taxa with differences in abundance between gestational age groups**

| **Gestational age and Taxa** | **Log fold change** | **P** | **Adjusted P*** |
| --- | --- | --- | --- |
| **Skin, Preterm > Term** |  |  |  |
| Uncultured bacterium* | 2.001 | <0.0001 | 0.0017 |
| Gammaproteobacteria | 1.881 | 0.0003 | 0.0054 |
| *Bacillus* | 1.665 | 0.0033 | 0.0292 |
| *Escherichia* | 1.291 | 0.0058 | 0.0439 |
| *Enterobacteriaceae* | 1.224 | 0.0019 | 0.0196 |
| *Staphylococcus* | 1.172 | 0.0002 | 0.0048 |
| *Enterobacter* | 1.169 | 0.0005 | 0.0061 |
| **Skin, Term > Preterm** |  |  |  |
| *Neisseria* | 1.734 | 0.0004 | 0.0054 |
| **Oral, Preterm > Term** |  |  |  |
| *Stenotrophomonas* | 2.103 | 0.0007 | 0.0213 |
| *Lactococcus* | 1.818 | 0.0021 | 0.0433 |
| *Enterobacter* | 1.631 | <0.0001 | 0.0013 |
| **Environment, Preterm > Term** |  |  |  |
| *Bacillus* | 3.349 | <0.0001 | <0.0001 |
| *Lysinibacillus* | 4.287 | <0.0001 | <0.0001 |
| Gammaproteobacteria | 3.616 | <0.0001 | 0.0005 |
| *Anaerobacillus* | 3.263 | <0.0001 | 0.0003 |
| Uncultured bacterium** | 2.866 | 0.0003 | 0.0030 |
| **Environment, Term > Preterm** |  |  |  |
| *Finegoldia* | 4.208 | 0.0006 | 0.0046 |

*False Discovery Rate (FDR) adjusted

**Class-*Gammaproteobacteria*, Order-*B38*, Family-uncultured bacterium
